# Synergistic effects of processing parameters on the biochemical and physical properties of tofu made from yellow field pea (*Pisum sativum*), as determined by response surface methodology

**DOI:** 10.1002/fsn3.2091

**Published:** 2021-01-06

**Authors:** Kevin DePalma, Brennan Smith, Armando G. McDonald

**Affiliations:** ^1^ Animal, Veterinary, and Food Science University of Idaho Moscow ID USA; ^2^ Department of Forest, Rangeland and Fire Sciences University of Idaho Moscow ID USA

**Keywords:** field peas (*Pisum sativum*), response surface, soy, soy‐free, tofu

## Abstract

Peas are an underutilized crop that do not require allergen labeling and are rarely genetically modified. Peas contain less protein than soy and vary in protein composition. Because peas contain more starch than soy and less lipids, an alternative procedure for pea tofu production needs to be developed to prevent excessive starch gelatinization while promoting curd development. To accomplish this, a response surface model design was utilized to determine optimal oil addition, cook time, and salt concentration. Treatment ranges were from 0.0% to 4.2% for oil addition, 60–134 min for cook time, and 5.0%–9.2% for MgCl_2_ addition. Treatments had varying effects on tofu texture. Cook time was directly proportional to the hardness and could be used to match the soft, firm, and extra firm texture targets of conventional soy tofu. Protein secondary structure was not related to gel strength, indicating a system with synergies between multiple components other than protein. This research will help satisfy the growing demand for alternatives to soy‐based foods.

## Graphical abstract

A variety of mechanical properties can be achieved with pea‐based tofu by altering cook time, ion concentration, and lipid concentration. Shifts in biochemical properties may not follow the same trend as hardness. Protein secondary structure was affected by experimental treatments but did not follow the same trends as hardness values.

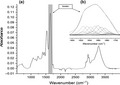

## 1 INTRODUCTION

Promotion of underutilized crops, allergenicity, and consumer mistrust over genetically modified organisms has resulted in a shift away from soy products to other legumes (Marcone et al., 1998). A meta‐analysis by Sicherer and York (2011) found that 1.4% of infants and between 0.1% and 0.7% of adults have allergic reactions to soy. Because soybeans are a subsidized crop that can be reliably grown, soy‐based ingredients have permeated the market, restricting options for those with soy allergies (DePalma et al., 2019). One product that has few nonsoy alternatives is tofu. Tofu has provided a historically accessible protein source. In current times, a soy‐free tofu would extend this protein accessibility to those with allergies and avoidance behavior to soy.

Glycinin and β‐conglycinin proteins account for 70% of the total protein in soy and are critical to the formation of tofu gels (Poysa et al., 2006). Glycinin forms a firm gel when unfolded by heat to expose cysteine, which forms intramolecular disulfide bridges and sulfur–hydrogen interactions (Phillips & Williams, 2011). While glycinin has between 10 and 15 cysteine residues, β‐conglycinin has two or three, forms gels with lower hardness values and more elasticity (Phillips & Williams, 2011). Because proteins from the yellow field pea (*Pisum sativum*) have similar biochemical composition to that of soy, it can be hypothesized that they will be able to coagulate, make a curd, and form a soy‐free tofu analog.

While tofu production can vary by manufacturer, the basic steps are soaking and grinding soybeans into a slurry, filtering the slurry to make soy milk, heating the milk, adding coagulants, and pressing the resulting curds. In traditional tofu, soy proteins need to dissociate to expose the hydrophobic regions. This occurs between 65 and 75°C depending on ionic strength, with higher ionic strength increasing the temperature required for dissociation of glycinin and β‐conglycinin in soy (Matsudomi et al., 1985). Other research found that some of these proteins can remain undissociated after 30 min (Wu et al., 2017), and pea proteins are more thermally stable, dissociating between 75 and 85°C (Mession et al., 2015). Because the change in dissociation temperature is dependent on ionic strength (Matsudomi et al., 1985), the degree of dissociation prior to coagulant addition will affect the amount of protein available to react with the coagulant. Unless the proteins are fully dissociated at the time of coagulant addition, excess coagulant is likely to result (Cai & Chang, 1998).

While there have been many studies on soy tofu and the underlying mechanisms required for its production, there has been very little published on production and quality of soy‐free tofu. The most comprehensive study reviewed five beans, chickpeas, lentils, smooth peas, mung beans, and fava beans (Cai et al., 2002). The methods called for extensive extraction and centrifugation, followed by an aggressive heating step, requiring 10 min of boiling. A study by Jayasena et al. (2010) looked to replace a certain amount of soy beans with lupin beans for lower fat and cost tofu. Up to 40% of the soy beans could be replaced with no significant effects to texture. Gebre‐Egziabher and Sumner (1983) produced tofu with pea flour and pea protein concentrate using calcium sulfate and found that an acceptable curd could be formed. However, the aforementioned studies did not look at industry‐friendly methods of tofu production.

In work by DePalma et al. (2019), a physical disruption of curds made from the coagulation of yellow field pea proteins with heat and MgCl_2_ produced a tofu analogue with similar characteristics as commercially available soy tofu. This study investigated quality, protein secondary structure, and microstructure of pea‐based tofu analogues in response to lipid level and physical disruption of protein curds followed by repressing. It also focused on individual variables and not the synergistic effects of multiple variables on tofu biochemical properties and resulting textures. Considering the affects ionic strength, the temperature required for dissociation, time‐temperature effects on protein dissociated, and the effect of oil concentration on protein dissociation and aggregation, there is an ample amount of synergistic interaction in the system. The objective of this study is to determine the synergistic effects of different processing factors (cook time, MgCl_2_, and oil concentration).

## 2 MATERIALS AND METHODS

### 2.1 Materials

Yellow split peas were obtained from Columbia Bean & Produce. The dried, refined Japanese Nigari (MgCl_2_) was purchased from Handy Pantry.

### 2.2 Response Surface Methodology

A variation of response surface methodology (RSM) used by Smith et al. (2019) and Smith et al. (2012) was used to determine the effects of cook time, MgCl_2_ addition, and oil addition. Preliminary tests were run to approximate the range of treatment levels to be used. A central composite design was prepared in Stat Ease 10 (Stat‐Ease Corporation). Five levels of cook times (45–150 min), MgCl_2_ (4.2%–10% [w/w]), and oil (0.0%–4.2% [w/w]) were chosen (Table 1), and 11 combinations of the variables at the selected levels were completed. Error was assessed using five replicates of one treatment combination. A second‐order polynomial regression model (Equation 1) was used to select linear, quadratic, or if there was interaction between variables (cross‐product) for analysis of variance (ANOVA).

(1) $$Y=\beta_{0}+\sum_{i=1}^{n}\beta_{i}x_{i}+\sum_{i=1}^{n}\beta_{\mathrm{ii}}x_{i}^{2}=\sum_{i,j=\left(i\neq j\right)}^{n}\beta_{\mathrm{ij}}x_{i}x_{j}$$

where *Y* is the response variable. When *i = j*, *β*_0_ is the coefficient of intercept, *β_i_* is the coefficient for linear, *β_ij_* is the coefficient for quadratic, and when *i ≠ j*, *β_ij_* is the cross‐product coefficient. $x_{i}$ and $x_{j}$ are terms used to describe the independent factors.

**TABLE 1:** Coded variable levels for time, MgCl2, and oil for the experimental RSM design

Experimental design			
Treatment	Time	Coded levels^a^	
		MgCl_2_	Oil
1	−1	+1	+1
2	0	0	0
3	−1.414	0	0
4	0	0	+1.414
5	+1.414	0	0
6	0	0	0
7	0	0	0
8	0	−1.414	0
9	0	0	0
10	0	0	0
11	−1	−1	+1
12	+1	−1	+1
13	+1	+1	−1
14	0	+1.414	0
15	0	0	−1.414

^a^ Coded levels: Time (min): −1.414 = 45 min, −1 = 60.38 min, 0 = 97.5 min, +1 = 134.62 min, +1.414 = 150 min; MgCl_2_ (w/w): −1.414 = 4.2%, −1 = 5.05%, 0 = 7.1%, +1 = 9.15%, +1.414 = 10%; oil (w/w): −1.414 = 0.0%, −1 = 0.62%, 0 = 2.1%, +1 = 3.58%, +1.414 = 4.2%. Percent values are (w/w).

The sequential model sum of squares (SMSS), lack‐of‐fit tests, and the multiple correlation coefficient (*R* ^2^) were used to select a model (mean = no model, linear, quadratic, or cross‐product) for each response. Significance was defined as *p* < .05.

### 2.3 Pea Milk and Tofu Preparation

Pea milk and pea‐based tofu were produced as described by DePalma et al. (2019) with changes. Pea milk (481 g) plus corn oil treatments (0.0%–4.2% [w/w]) were added to a 1,000‐ml beaker. The mixture was blended with a Cuisinart immersion blender for 2 min to homogenize. The beaker was placed in a 98°C water bath for the duration of a given cook time (45–150 min). After heating, MgCl_2_ (4.2%–10% [w/w]) was added over the course of 10 s, stirring constantly. After 10 min, the curds were poured into a 12.8 × 9.1 × 7.9 cm tofu mold lined with a cotton cloth. The cloth was folded once over the curds, and a perforated piece of plastic was placed on the cloth before the other sides of the cloth were folded over to prevent molding defects. A 5 kg weight was placed on top of the mold for 10 min. The pea tofu was removed from the mold and cooled for 10 min before being wrapped in plastic wrap and refrigerated.

An additional sample set of pea‐based tofu was prepared in duplicate using the method previously described. The tofu was cut into strips and placed into a 50‐mL centrifuge tube. The tube was submerged in liquid nitrogen until tofu was frozen. The samples were held in a −80°C freezer until being freeze‐dried in a FreeZone 6 (Labconco) at −56°C and 0.02 mbar. The freeze‐dried samples were stored in a −80°C freezer until biochemical analysis was performed.

### 2.4 Physical Properties of Tofu

Samples were equilibrated to room temperature for 30 min prior to testing. Two blades fastened together 10 mm apart were used to cut the samples into 10 mm cubes. A 25‐mm‐diameter cylindrical plastic probe was attached to a TA.XT.plus texture analyzer (Stable Micro Systems Ltd.) equipped with a 5 kg load cell. Texture profile analysis (TPA) was run with a pretest speed of 1.00 mm/s, test speed of 0.83 mm/s, posttest speed of 2.00 mm/s, a trigger force of 5.0 g, a compression strain of 30% of the block thickness, and a 2 s rest between the compressions.

Tofu color was assessed by L*, a*, and b* color parameters according to the CIELAB international system of color measurement. To accomplish this, a piece of tofu larger than the 8‐mm‐diameter colorimeter aperture was placed on Konica Minolta Spectrophotometer CM‐5 (Konica Minolta Sensing Americas, Inc.) equipped with an 8 mm aperture and instrument set to D65 with a 10% light angle. Excess moisture was blotted from the surface of the sample prior to analysis.

### 2.5 Percent Yield

The mass of the block of tofu was measured on a Mettler Toledo New Classic SG digital balance. Percent yield was determined by Equation 2:

(2) $$\left(\text{Mass of tofu}/\text{mass of pea milk}+\text{corn oil}\right)\times 100=\%\;\text{yield}$$

### 2.6 Percent Retained Solids

Retained solids are the percent of solids that went into the system and were not pressed out. The value was calculated by the following equation:

(3) $$\left(\text{tofu solids}\;\left(g\right)‐\text{oil added}\;\left(g\right)\right)/\left(\text{pea milk solids}\;\left(g\right)+{\text{MgCl}}_{2}\text{solids}\;\left(g\right)\right)\times 100\;=\;\%\;\text{retained solids}$$

### 2.7 Optimization

Optimization of desirability was determined by the equation as described by Smith et al. (2019):

(4) $$D={\left(d_{1}^{r_{2}}\times d_{2}^{r_{2}}\times \ldots d_{n}^{r_{n}}\right)}^{\frac{1}{\sum r_{i}}}$$

where *D* represents desirability; *d_i_* is the desirability for each response; and *r_i_* is relative importance (with 1 being most desirable, *D* values can range from 0 to 1).

### 2.8 FT‐IR Spectroscopic Analysis

Freeze‐dried samples were equilibrated to room temperature for 45 min prior to obtaining spectra on a Nicolet iS10 Fourier transform IR spectrometer (Thermo Fisher Scientific) that was purged continuously with dry air and equipped with a diamond attenuated total reflection accessory (Smart orbit, single bounce, 45°), which was used to measure the IR spectra. FT‐IR spectral analysis was performed as described by DePalma et al. (2019). The spectra for added oils were subtracted prior to baseline correction and deconvolution.

### 2.9 Surface Hydrophobicity

A modified method of Chelh et al. (2006) was used to measure surface hydrophobicity by spectrophotometry at 595 nm after addition of bromophenol blue (BPB) as described by DePalma et al. (2019).

### 2.10 Molecular Weight Analysis

Freeze‐dried ground sample (0.5 g) was placed in centrifuge tubes (50 ml) and vortexed at 450 rpm for 30 min with an excess of hexane and centrifuged for 1 min at 340 ***g***. The supernatant (lipids) was decanted, and remaining hexane was evaporated in open air. Removal of lipids improved the resolution of the electropherograms.

Extractions were carried out as described by DePalma et al. (2019) and analyzed with an Agilent Bioanalyzer 2100 (Agilent).

### 2.11 Microscopy

A piece of freeze‐dried sample, approximately 2 mm × 2 mm, was imaged by scanning electron microscopy (SEM) with backscattering electrons (BSE) on a Quanta 200F SEM (FEI Company) at 130 Pa and an accelerating voltage of 15.0 kV.

## 3 RESULTS AND DISCUSSION

### 3.1 Physical Properties of Tofu

**FIGURE 1:**
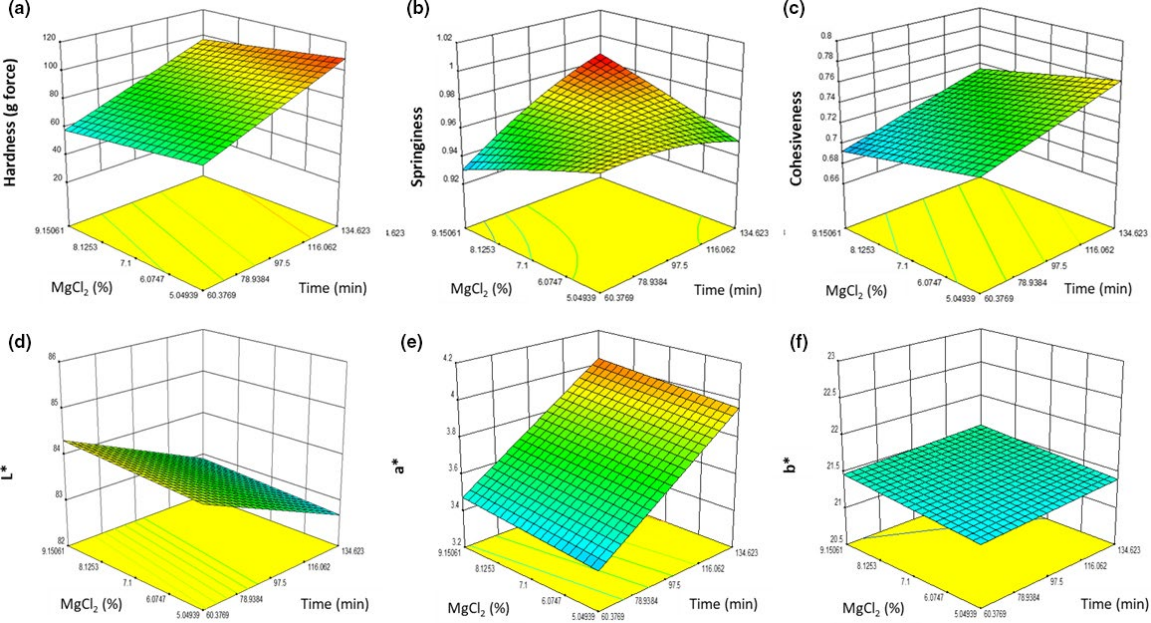
A graphical representation of the effect of present MgCl_2_ (w/w) (*y*‐axis) and time (*x‐*axis) at 2.14% (w/w) oil addition on the physical properties of pea‐based tofu with a) hardness, b) cohesiveness, and c) springiness on the vertical axis (top row) and tofu color, d) L*, e) a*, and f) b* on the vertical axis (bottom row)

The properties (hardness, springiness, cohesiveness, and color coordinates) of the tofu were optimized using an RSM for cook time and MgCl_2_ content. The linear model for hardness (Figure 1a) was significant (*p* = .0165). The hardness of the tofu was significantly affected by cook time (*p* = .0049), increasing the hardness by 50 g between 60 and 134 min when all other variables were held constant. The hardness was not significantly affected by = MgCl_2_ content (*p* = .3175), reducing the hardness by 10 g between 5.05% and 9.15% (w/w) when all other variables were held constant. The hardness was not significantly affected by oil addition (*p* = .1397), decreasing the hardness by approximately 20 g between 0.62% and 3.55% (w/w) oil.

The quadratic model for springiness (Figure 1b) was not significant (*p* = .2672). The springiness of the tofu was not significantly affected by cook time (*p* = .1836), MgCl_2_ content (*p* = 1.0000), or oil addition (*p* = .2336). Interactions between the factors reduce predictability of individual factors.

The linear model for cohesiveness (Figure 1c) was significant (*p* = .0199). The cohesiveness of the tofu was significantly affected by cook time (*p* = .0224), increasing the cohesiveness by 0.02 between 60 and 134 min when all other variables were held constant. The cohesiveness was not significantly affected by MgCl_2_ content (*p* = .1135), reducing the cohesiveness by 0.07 units between 5.05% and 9.15% (w/w) when all other variables were held constant. The cohesiveness was significantly affected by oil addition (*p* = .0471), decreasing the cohesiveness by 0.04 units between 0.62% and 3.55% (w/w) oil.

The cook time is the only significant factor in determining the hardness, and one of two significant factors affecting cohesiveness. With heating, soy proteins have been shown to not immediately dissociate, with some remaining undissociated after 30 min of heating (Wu et al., 2017). This phenomenon could be exacerbated in pea proteins, which require 75–85°C to dissociate, as opposed to soy that only requires 65–75°C to dissociate (Mession et al., 2015; Wu et al., 2017).

The MgCl_2_ content was not a significant factor for any mechanical property tested. However, the rate of coagulant addition has been shown to affect the mechanical properties of soy tofu (Cai & Chang, 1998). In this study, the concentration of MgCl_2_ was adjusted by changing the amount of the stock MgCl_2_ solution. While the duration of MgCl_2_ addition was constant for all samples (10 s), the amount was not. This means the rate of MgCl_2_ addition varied between treatments. This could potentially have led to confounding variables but was unavoidable given the needed treatment variables.

The addition of oil did not significantly affect the hardness, which varied from previous work that showed a significant decrease (DePalma et al., 2019). This study also investigated pea‐based tofu made under different processing conditions as investigated here (i.e., lower processing temperatures). Since the RSM model shows a trend of decreasing hardness with oil addition, the significance of the oil addition is likely lost in the noise of the MgCl_2_ addition and significance of the heat treatment. The addition of oil also affected the cohesiveness, which is likely because the protein content in the resulting tofu decreased with oil addition. Hypothetically, this would shift the balance away from the gel‐forming component and toward a liquid. Although it is plausible, this decrease is partially caused by redistribution of protein subunits, as shown by the surface hydrophobicity assay discussed later. Overall, MgCl_2_ concentration and oil level decreased hardness. However, the decrease in these values were overcome by increasing cook time. It should also be mentioned that in the study by DePalma et al., (2019), a coagulation temperature common in soy tofu manufacturing (80°C) was used. In the current study, 98°C was selected while determining the anchor points for the RSM design, as improvement in texture could not be captured with reasonable time treatments at 80°C. This demonstrates that an increased MgCl_2_ concentration and more severe heat treatments alone can accomplish similar results as disrupting and repressing yellow pea tofu curds as described by DePalma et al. (2019), providing yet another feasible method to produce tofu analogs from yellow peas.

The linear model for L* value (Figure 1d) was significant (*p* = .0190). The L* value of the tofu was significantly affected by cook time (*p* = .0056), decreasing the L* value by 1.2 units between 60 and 134 min when all other variables are held constant. The *L value was not significantly affected by the MgCl_2_ content (*p = .4689*), exhibiting no change in the *L value between 5.05% and 9.15% (w/w) when all other variables were held constant. The L* value was not significantly affected by oil addition (*p* = .1147), increasing the L* value by approximately 0.8 units between 0.62% and 3.55% (w/w).

The decrease in lightness associated with cook time is likely due to decreased water content, resulting in an increased concentration of pigments and a darker color. However, it should be mentioned that when samples with no additional oil were compared to samples with added oil, the samples with added oil were visibly a lighter color. Therefore, these results indicated that the amount of oil added does not increase the L* after a certain addition level.

The linear model for a* value (Figure 1e) was significant (*p* = .0032). The a* value of the tofu was significantly affected by cook time (*p* = .0004), decreasing the a* value by approximately 0.6 units between 60 and 134 min when all other variables were held constant. The a* value was not significantly affected by the MgCl_2_ content (*p* = .3397), exhibiting no change in the a* value between 5.05% and 9.15% (w/w) when all other variables are held constant. The a* value was not significantly affected by oil addition (*p* = .9424), exhibiting no change in the a* value between 0.62% and 3.55% (w/w) when all other variables were held constant.

The 2FI (two‐factor interaction) model for b* value (Figure 1f) was significant (*p* = .0049). The b* value of the tofu was not significantly affected by cook time (*p* = .6500), decreasing the b* value by 1.2 units between 60 and 134 min when all other variables were held constant. The b* value was not significantly affected by the MgCl_2_ content (*p* = .6127), exhibiting no change in the b* value between 5.05% and 9.15% (w/w) when all other variables were held constant. The b* value was significantly affected by oil addition (*p* = .0222), increasing the b* value by approximately 0.8 units between 0.62% and 3.55% (w/w) oil addition levels.

In these data, the positive a* indicates a very slight redness, but the predominating color is yellow, as indicated by the b* values. Tofu prepared from yellow split peas had a more yellow appearance than traditional soy tofu, which could be considered a product defect (Kim and Wicker, 2005). Fortunately, the yellow hue can be mitigated through the addition of oil and modification of heat treatments (Figure 1a–f). For reference, Kim and Wicker (2005) reported L* values of 90 and 93 for soy tofu, while the pea tofu in this study ranged from 82 to 85.

### 3.2 Percent Yield

**FIGURE 2:**
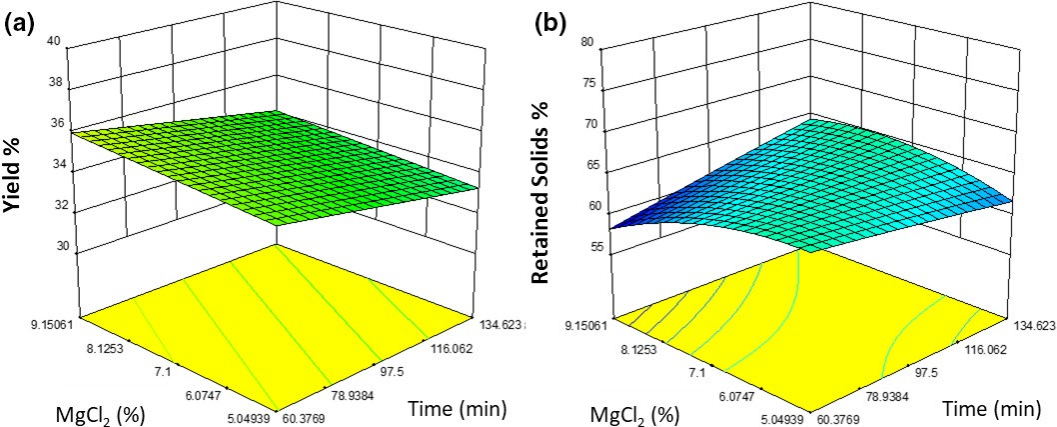
A graphical representation of the effect of percent MgCl_2_ (w/w) (*y*‐axis) and time (*x*‐axis) at 2.14% (w/w) oil addition with on (a) percent yield and (b) percent retained solids

The linear model for percent yield (Figure 2a) was significant (*p* = .0316). The percent yield of the tofu was not significantly affected by cook time (*p* = .2124), decreasing the percent yield by approximately 3% (w/w) between 60 and 134 min when all other variables were held constant. The percent yield was not significantly affected by the MgCl_2_ content (*p* = .5353), increasing the percent yield by approximately 1% (w/w) between 5.05% and 9.15% (w/w) when all other variables were held constant. The percent yield was significantly affected by oil addition (*p* = .0076), increasing the percent yield by approximately 4% (w/w) between 0.62% and 3.55% (w/w) addition levels. The percent yield was most affected by oil addition, which is to be expected, since the oil is incorporated into the gel and the bulk of the water was removed during pressing.

### 3.3 Percent Retained Solids

While percent yield is important, greater attention must be given to the percent retained solids. This value represents additional nutrients originating from the raw peas that get incorporated into the tofu gel, resulting in less loss for the manufacturer. The quadratic model for percent retained solids (Figure 2b) was significant (*p* = .0075). The retained solids of the tofu were not significantly affected by cook time (*p* = .3133), and retained solids were not significantly affected by the MgCl_2_ concentration (*p* = .1763). There was an interaction between cook time and MgCl_2_ concentration; at low MgCl_2_ concentrations, retained solids were decreased by 3% (w/w), and at high MgCl_2_ concentrations, retained solids were increased by 6% (w/w). The retained solids were significantly affected by oil addition (*p* = .0027), increasing the retained solids by approximately 6% (w/w) between 0.62% and 3.55% (w/w).

It is important to note that retained solids accounted for only the solids from the pea milk and MgCl_2_. Since the oil is completely incorporated by the curds, the only solids that can be removed during pressing come from the pea milk and MgCl_2_. Therefore, it is important to focus on how much of the pea solids are lost and MgCl_2_ is left, because it is difficult to determine how much of this salt is incorporated. The oil addition had little effect on retained solids at the lower levels. However, after a retained solids value of 2.4% (w/w) was reached, the values increase rapidly, as it was a quadratic function. This likely represents a critical value of when proteins with higher binding affinities to lipids become saturated and excess oil is free to bind to unassociated protein subunits.

### 3.4 Optimization

For this research, the optimal quality tofu was one considered to have the highest hardness values, greatest percent yield and retained solids, and the highest L* value. When weighted equally within the model, optimizing the aforementioned values resulted in predicted treatments of 134.6 min cook time, 7.961% MgCl_2_, and 2.1% oil. Here, a desirability was of 0.527 with a hardness of 98.62 g force, L* of 82.84, percent retained solids of 65.03, and a percent yield of 34.16. Due to the inverse relationship of some of the responses in regard to quality, the lower desirability value is to be expected. When oil addition is increased, greater L* values, yield, and retained solids could be realized. However, increasing oil decreased the gel hardness in this study. To this end, hardness is considered the most important quality factor in tofu (DePalma et al., 2019). Therefore, if all other factors are ignored and the tofu was optimized based on hardness alone, a 1.0 desirability value was achieved, with the model predicting a hardness value of 112.13 g force. For future work, or in application in industrial settings, one must set the lowest limits for what are acceptable values for yield, percent retained solids, and L* to meet the manufacturing and consumer needs, while maximizing the hardness of the pea tofu analog.

### 3.5 FT‐IR Spectroscopic Analysis

**FIGURE 3:**
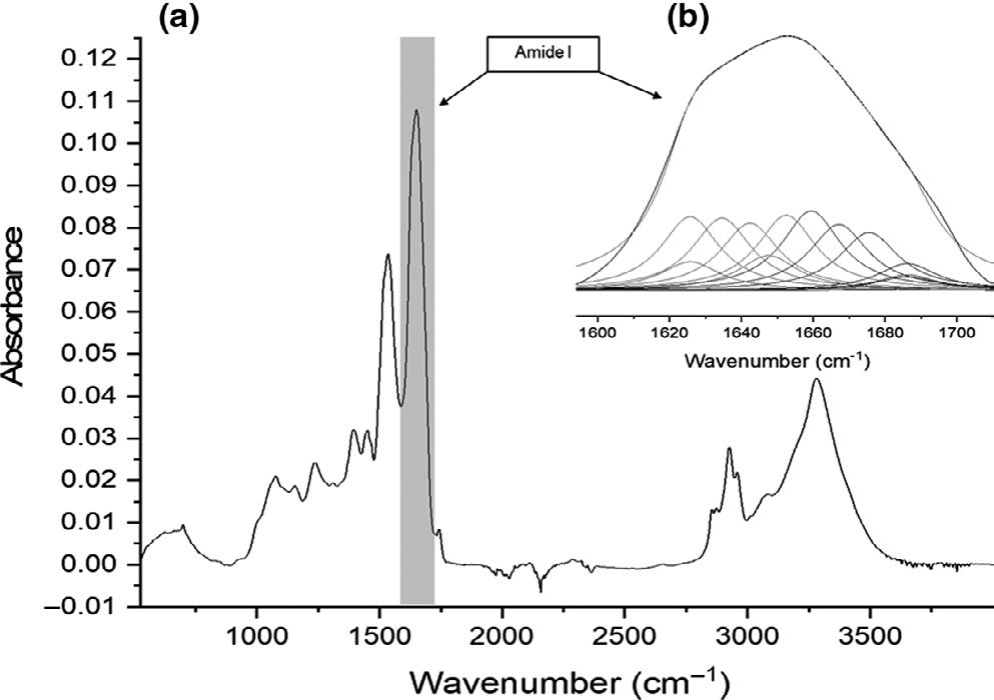
A representative ATR‐FTIR spectra of experimental pea‐based tofu, where (a) is the spectra between wave number 4,000 and 500 cm^−1^, with the wave number (1,720 and 1,580 cm^−1^) of the spectra containing the amide I region highlighted, and b) represents an expanded view, demonstrating the deconvolution of the amide I region

FT‐IR spectroscopy can provide information on protein structure in pea tofu. The range of 1,700‐–600 cm^−1^, or amide I band, was deconvoluted, and the percent of the amide I bands comprised of α‐helices (~1654 cm^−1^) and β‐sheets (~1636 cm^−1^; Shevkani et al., 2015) was determined (Figure 3). The areas in the amide I region associated with unordered structures were found to be too variable to effectively assess (data not shown). This is a common issue, due to the overlapping of hidden bands in the upper and lower sections of the amide I region (DePalma et al., 2019).

**FIGURE 4:**
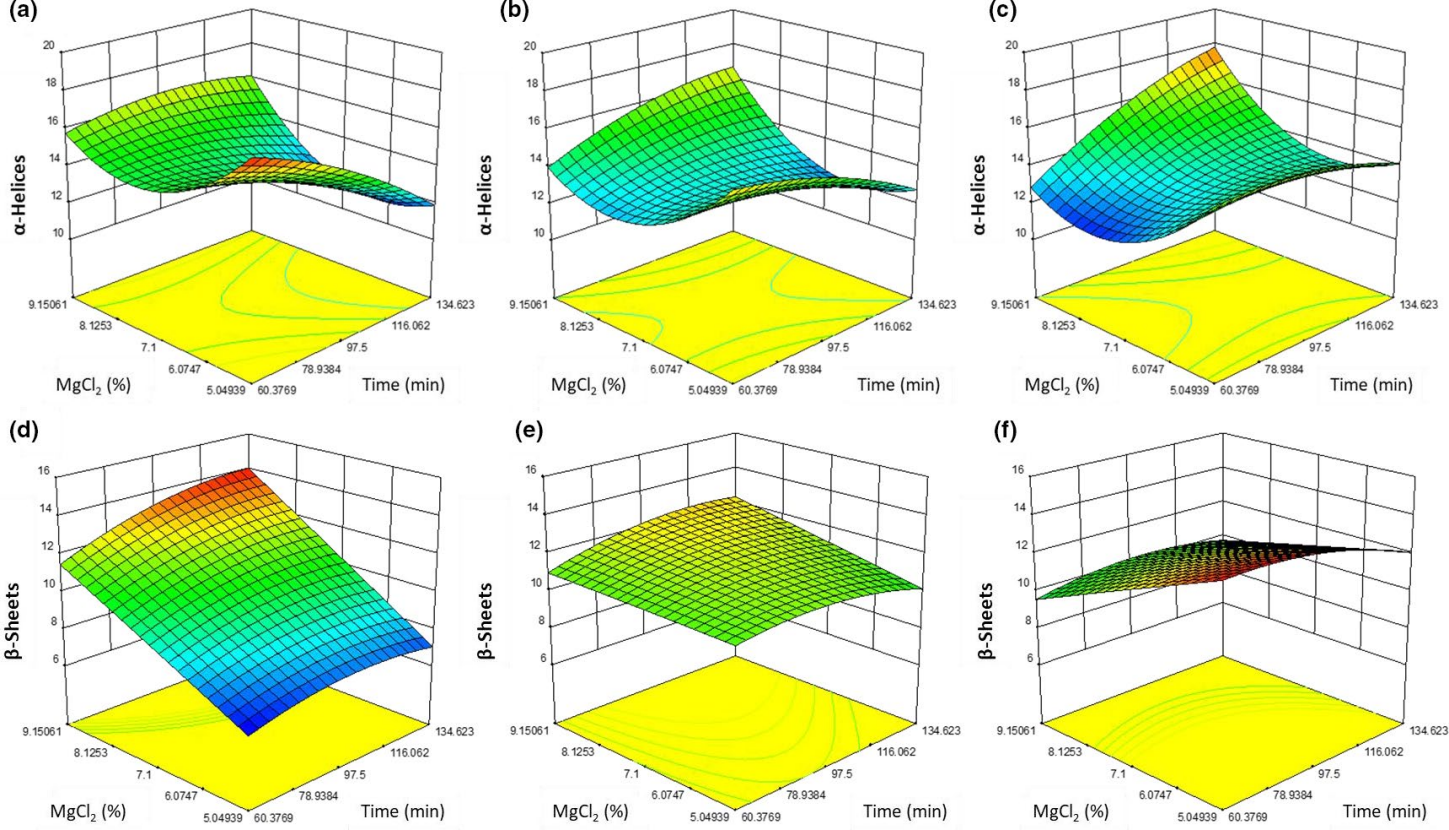
A graphical representation of the effect of percent MgCl_2_ (w/w) (*y*‐axis) and time (*x*‐axis) at (a) 0.62% (w/w), (b) 2.14% (w/w), and (c) 3.55% (w/w) oil addition with the vertical axis being α‐helices (top row) and the effect of percent MgCl_2_ (w/w) (*y*‐axis) and time (*x*‐axis) at (d) 0.62% (w/w), (e) 2.14% (w/w), and (f) 3.55% (w/w) oil addition with the vertical axis being β‐sheets (bottom row)

For the pea‐based tofu in this study, the quadratic model for α‐helices at different oil contents (Figure 4a–c) was significant (*p* = .0252). The α‐helices of the tofu were not significantly affected by cook time (*p* = .6790, MgCl_2_ content (*p* = .4520), or oil addition (*p* = .4520) alone. However, the interaction between time and MgCl_2_ content (*p* = .0824) and the quadratic value of oil addition (*p* = .0014) had significant effects.

The quadratic model for β‐sheets at different oil contents (Figure 4d–f) was significant (*p* = .0406). The β‐sheets of the pea‐based tofu proteins were not significantly affected by cook time (*p* = .7054), MgCl_2_ content (*p* = .2189), or oil addition (*p* = .1573) alone. However, the interaction between MgCl_2_ and oil addition (*p* = .0162) did have a significant affect; at low levels of oil addition, the percent of β‐sheets increased from 7% to 14% between the MgCl_2_ concentration of 5.0 and 9.2% (w/w) and the maximum cook time. At high levels of oil addition, β‐sheets decreased from 13% to 10% between the MgCl_2_ concentration of 5.0 and 9.2% (w/w) at the maximum cook time. This may be related to ionic‐mediated dissociation of vicilin and legumin proteins (Matsudomi et al., 1985). For this reason, the dissociation of the proteins was likely related to the low percent of β‐sheets in solutions of low ionic strengths. Since β‐sheets correlate with retained solids, it is possible that the solids being retained were high in β‐sheets and would thus be protein.

### 3.6 Surface Hydrophobicity

**FIGURE 5:**
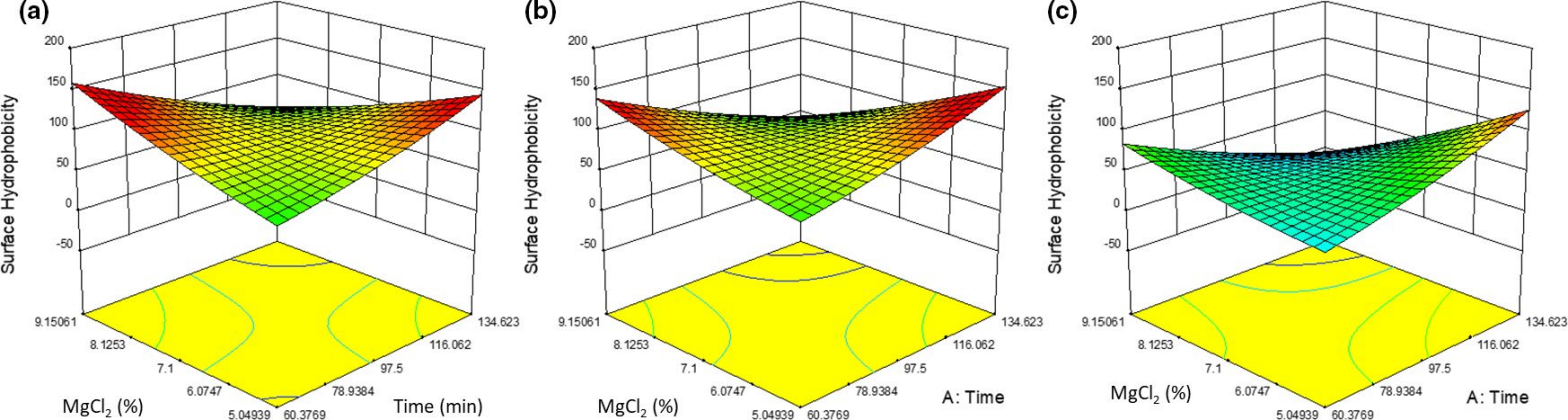
A graphical representation of the effect of percent MgCl_2_ (w/w) (*y*‐axis) and time (*x*‐axis) on surface hydrophobicity (µg of bound bromophenol blue) (vertical axis) with oil at a) 0.620%, (b) 2.14%, and (c) 3.55%

The quadratic model for surface hydrophobicity (Figure 5a–c) was significant (*p* = .0074). The surface hydrophobicity of the tofu was not significantly affected by cook time (*p* = .3269). The surface hydrophobicity was significantly affected by the MgCl_2_ content (*p* = .0498). The cook time and MgCl_2_ content did have an interaction with oil levels; at low oil concentrations, surface hydrophobicity was directly proportionate to cook time and MgCl_2_ concentrations, but at high oil levels the surface hydrophobicity was inversely proportionate to cook time and MgCl_2_ concentrations (Figure 5c). The surface hydrophobicity was significantly affected by oil addition (*p* = .0038), decreasing by approximately 25–50 µg BPB between 0.62% and 3.55% (w/w) oil if all other factors were held constant.

At low ionic strength, cook time increased the surface hydrophobicity, and at high ionic strength, cook time decreased surface hydrophobicity. Lower ionic strength is known to promote aggregation of legumin (Gueguen et al., 1988), so there should be less dissociation and thus less exposure of the hydrophobic interior regions of proteins, resulting in less binding of BPB. At low oil concentrations, the cook time increased the surface hydrophobicity, but at higher oil concentrations, cook time decreased the surface hydrophobicity. As the vicilin and legumin are heated, they dissociate into subunits, decreasing molecular weight and exposing hydrophobic regions (Mession et al., 2015). With oil addition, the newly exposed hydrophobic regions should associate with the lipids. This was seen in the decrease in surface hydrophobicity as the samples were cooked longer, with higher ionic strengths and oil addition. The change in the trend from increasing to decreasing surface hydrophobicity when oil levels are increased (Figure 5a–c) is likely from newly exposed hydrophobic surfaces on the dissociated protein subunits coating the surface of oil globules (Mession et al., 2015).

In previous studies, secondary structure, α‐helices and β‐sheets, is often proportional to surface hydrophobicity and textural properties of tofu (DePalma et al., 2019). Within these studies, it is typical to have one or two variables and measure the treatment's effect on the resulting gel. It is often found that protein secondary structures are proportional to gel textural properties. In this study, exposure to heat, ion concentration, and oil addition were studied. It was found that treatment effects were not necessarily proportional to resulting protein secondary structure data. The findings of this study suggest that the synergy between treatments has a large influence on protein secondary structure, surface hydrophobicity, and gel strength. This phenomenon is further compounded by the fact that in foods and other biological materials, the material being studied is rarely comprised of a single component. In this study, the treatments likely interacted with other endogenous components such as starch, polar lipids, sugars, and nonstarch polysaccharides to affect gel strength. However, from this work, it is evident that quantification of α helices and β sheets alone may not be a good indicator of gel strength and does not necessarily have to correlate with gel textural properties. To this end, care must be taken in subsequent studies to ensure that protein structure is not falsely correlated with gel textural properties and casual relationships must be demonstrated.

### 3.7 Molecular Weight Analysis

While field peas differ from soybeans in many aspects, the major protein classes of field peas are structurally analogous to glycinin and β‐conglycinin of soy. Glycinin and β‐conglycinin proteins account for 70% of the total protein in soybeans and are critical to the formation of tofu gels (Poysa et al., 2006). Glycinin forms a firm gel when unfolded by heat to expose cysteine, which forms intramolecular disulfide bridges (Phillips & Williams, 2011). While glycinin has between ten and 15 cysteine residues, β‐conglycinin has between two and three and forms gels with lower hardness values and more elasticity (Phillips & Williams, 2011). The legumin fraction in peas is similar to the glycinin fraction in soy, each being a 360 kDa hexamer (Phillips & Williams, 2011; Schwenke et al., 2001) comprised of acidic and basic subunits (Bacon et al., 1987). The key difference is that glycinin is held together with disulfide bonds that form interprotein cross‐linkages, while the legumin fraction is held together by strong hydrophobic interactions (Phillips & Williams, 2011). In systems with high hydrophobicity, protein–protein interactions are more abundant than protein–solvent interactions resulting in coagulum rather than formation of a true gel (Phillips & Williams, 2011). However, legumin has around 63% of the cysteine as glycinin (O’Kane et al., 2004a), so disulfide bonds may still form during aggregation to form curds similar to that of soy. Furthermore, when glycinin gels have been exposed to 2‐mercaptoethanol, they were only partially dissolved, indicating the importance of hydrophobic interactions (Nakamura et al., 1984). It seems likely that both glycinin and legumin gels are formed through disulfide bonds of cysteine residues and hydrophobic interactions between acidic and basic subunits. Soy β‐conglycinin and pea vicilin are trimetric proteins of ~170 kDa (Maruyama et al., 1999; O’Kane et al., 2004b). Therefore, understanding the relationship between processing perimeters, pea tofu gel physical properties, and protein molecular weight distribution is critical.

**FIGURE 6:**
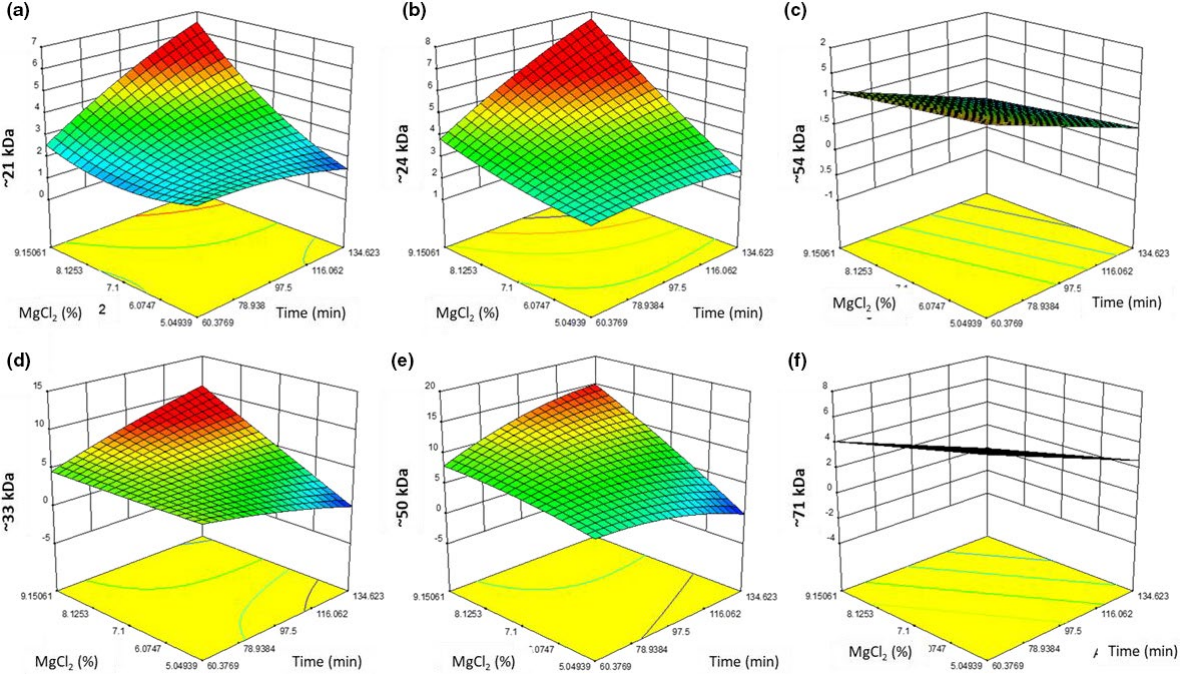
A graphical representation of the effect of percent MgCl_2_ (w/w) (*y*‐axis) and Time (*x‐*axis) at 2.14% (w/w) oil addition with the vertical axis being (a) ~21 kDa, (b) ~24 kDa, (c) ~54 kDa, (d) ~33 kDa, (e) ~50 kDa, and (f) ~71 kDa

The model for the ~21 kDa peak (Figure 6a) was not significant (*p* = .6912). The ~24 kDa peak of the tofu was not significantly affected by cook time (*p* = .6951), MgCl_2_ (*p* = .8952), or oil addition (*p* =0 .5566).

The quadratic model for the ~24 kDa peak (Figure 6b) was not significant (*p* = .3449). The ~24 kDa peak of the tofu was not significantly affected by cook time (*p* = .8578), MgCl_2_ content (*p* = .5219), or oil addition (*p* = .2670). The most significant component of the model (*p* = .0582) was the effect of the oil squared, which contributed to the drastic increase in the amount of the ~24 kDa polypeptide. The ~24 kDa peak is a polypeptide of legumin. The ~24 kDa peak increasing with time at higher oil levels could indicate that as legumin broke‐down into its base components, the ~24 kDa polypeptide was retained by the oil, contributing to the retained solids (Figures 2b and 6b).

The linear model for the ~54 kDa peak (Figure 6c) was not significant (*p* = .0761), nor was it significantly affected by MgCl_2_ (*p* = .4190) or oil addition (*p* = .5192). However, the ~54 kDa peak area was significantly affected by time (*p* = .0170), decreasing by approximately 1.5% (w/w) between 60 and 134 min when all other variables are held constant.

One of the key proteins in peas is legumin. The ~21 and ~24 kDa polypeptides are two of the alkaline polypeptides that make up a legumin subunit. Legumin breaks down into subunits that are reported to be approximately between 54 and 60 kDa (Schwenke et al., 1990). The breakdown of the ~54 kDa protein into the ~21 and ~24 kDa is consistent with glycinin of soy breaking into subunits with heating during soy tofu production. These data also indicate that pea‐based tofu follows a similar mechanism of formation as conventional soy tofu as outlined above. This hypothesis was further supported by the surface hydrophobicity data that demonstrated an increase in dissociation with increased cook time (Figures 5a–c and 6c). The amount that cook time increased the ~21 kDa and the ~24 kDa peak areas increased with oil addition. However, oil addition did not affect the ~54 kDa peak as much, indicating that legumin's breakdown was not augmented by oil addition, but the retention of ~21 and ~24 kDa polypeptides in the pea tofu was.

The other critical protein in the pea tofu system was vicilin, which is comprised of subunits of ~33, ~50, and ~71 kDa. The quadratic model for the ~33 kDa (Figure 6d) was not significant (*p* = .3262). The ~33 kDa of the tofu was not significantly affected by cook time (*p* = .4447), MgCl_2_ (*p* = .6695), or oil addition (*p* = .4023). The change in the ~33 kDa was difficult to interpret, as the ~50 kDa subunit is comprised in part of a polypeptide that is also ~33 kDa. The quadratic model for the ~50 kDa (Figure 6e) was significant (*p* = .0334). The ~50 kDa peak of the extracted pea tofu proteins was not significantly affected by cook time (*p* = .8635), MgCl_2_ (*p* = .4259), or oil addition (*p* = .2310) alone. However, the combined effect of the MgCl_2_ and oil addition was significant (*p* = .0178), as well as the quadratic effect of the oil addition (*p* = .0089). The ~33 and ~50 kDa peaks follow the same trend as the ~21 and ~24 kDa peaks, where cook time increased peak area more at high oil addition. The linear model for the ~71 kDa (Figure 6f) was not significant (*p* = .0603). The ~71 kDa of the tofu was significantly affected by cook time (*p* = .0244), reducing the ~71 kDa by 2% (w/w) between 60 and 134 min when all other variables were held constant. The ~71 kDa was not significantly affected by the MgCl_2_ (*p* = .1228), reducing the ~71 kDa by 2% (w/w) between 5.05% and 9.15% (w/w) MgCl_2_ addition level when all other variables were held constant. The ~71 kDa protein was not significantly affected by oil addition (*p* = .5415). The decrease in the ~71 kDa, a subunit of vicilin, was inversely proportionate to time and thus hardness, likely indicating that the ~71 kDa subunit was associating with other proteins during the formation of pea tofu gel.

### 3.8 Microscopy

**FIGURE 7:**
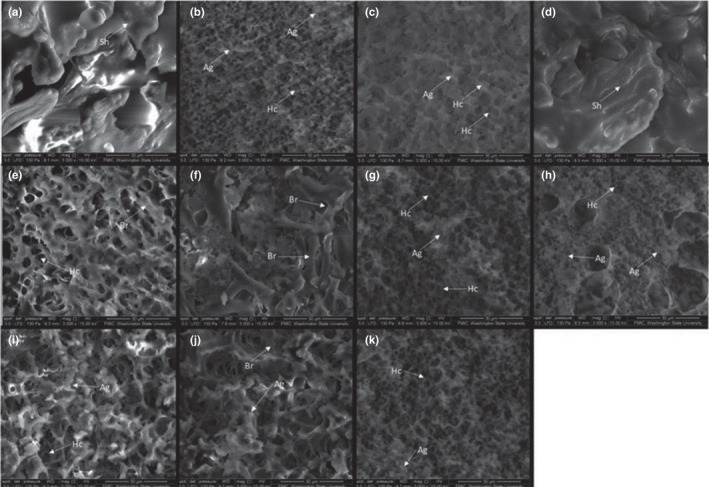
*SEM* of RSM treatments (a) 1 (−1, +1, 0), (b) 3 (−1.414, 0, 0), (c) 4 (0, 0, +1.414), (d) 5 (+1.414, 0, 0), (e) 7 (0, 0, 0), (f) 8 (0, −1.414, 0), (g) 11 (−1, −1, +1), (h) 12 (+1, −1, +1), (i) 13 (+1, +1, −1), (j) 14 (0, +1.414, 0), (k) 15 (0, 0, −1.414). All micrographs were taken at 3,000× magnification. Ag designates aggregates, Br designates branches, Hc designates honeycomb structures, and Sh designates sheets

All but two SEM images (Figure 7a,d) displayed honeycomb structures, a common structure reported in soy tofu (DePalma et al., 2019). The voids in the honeycombs increased as cook time increased, with the smallest voids in the 45 min cook time (Figure 7b) and progressively larger voids in the 60 min (Figure 7g), 97.5 min (Figures 7c,e,f,j,k), and 134 min (Figure 7h,i). The longest cook time, 150 min (Figure 7d), did not form honeycombs. Since heat time was found to correlate with pea tofu hardness, this is unexpected since previous work showed tofu with denser honeycombs and smaller voids had higher hardness values (DePalma et al., 2019). As defined by the presence of smaller voids, shorter cook times resulted in denser structures. However, as cook time increased, the branches that connected the aggregates became thicker, to the point where aggregates were no longer discrete.

## 4 CONCLUSION

The treatment plan demonstrated a range of textures for pea‐based tofu could be obtained by varying cook time, MgCl_2_, and oil content. Cook time was the most important factor for determining hardness, the most important quality characteristic of tofu. Furthermore, it was determined that the synergistic effects between treatments influenced gel strength and protein secondary structure. However, it was found that gel strength did not necessarily have to correlate with any one factor. The biochemical assays showed that there is thermal dissociation of legumin and vicilin, which forms a matrix that is affected by hydrophobic interactions. These interactions allowed for the incorporation of lipid to make a visibly smoother, lighter colored pea‐based tofu analog. These data can act as a template by manufacturers looking for a low‐cost, high‐protein, soy‐free food.

## AUTHOR CONTRIBUTIONS

K. DePalma designed and conducted research, analyzed data, and prepared manuscript. B. Smith designed and conducted research, analyzed data, and prepared manuscript. A.G. McDonald helped conduct research, analyze data, and aided in the preparation of the manuscript.
